# Adherence to the Mediterranean diet in young male soccer players

**DOI:** 10.1186/s40795-023-00761-6

**Published:** 2023-09-04

**Authors:** César Leão, Sílvia Rocha-Rodrigues, Inês Machado, João Lemos, Sandra Leal, Hadi Nobari

**Affiliations:** 1 Escola Superior Desporto e Lazer, Instituto Politécnico de Viana do Castelo, Rua Escola Industrial e Comercial de Nun’Álvares, Viana do Castelo, 4900-347 Portugal; 2 Research Center in Sports Performance, Recreation, Innovation and Technology (SPRINT), Viana do Castelo, Melgaço, 4960-320 Portugal; 3 Tumour & Microenvironment Interactions Group, INEB- Institute of Biomedical Engineering, i3S-Instituto de Investigação e Inovação em Saúde, Universidade do Porto, Rua Alfredo Allen, Porto, 4200-153 Portugal; 4 Instituto Universitário de Ciências da Saúde, Rua Central de Gandra, Paredes, 4585-116 Portugal; 5 Faculty of Sports Science, University of Extremadura, Cáceres, Spain; 6 Department of Exercise Physiology, Faculty of Educational Sciences and Psychology, University of Mohaghegh Ardabili, Ardabil, 5619911367 H.N Iran

**Keywords:** Anthropometry, Youth, Nutrition; Mediterranean, Team sports

## Abstract

**Introduction:**

Nutrition is vital in health and sports performance by improving anthropometric-related parameters and dietary habits, especially in the youngest ages. The Mediterranean diet (MD) has been highly recognized for its positive health effects and low adverse environmental impact.

**Objectives:**

We aimed to characterize adherence to the MD and analyze its association with anthropometric parameters in young soccer players.

**Methodology:**

In the present study, 132 male young soccer players from under 9 to under 15 categories (aged 7 to 15 years) from a Portuguese football club participated. The Mediterranean Diet Quality Index for Children and Adolescents (KIDMED) questionnaire was applied to assess adherence to the MD. Anthropometric-related parameters, including body mass, height, triceps skinfold thickness (TSKF), suprailiac skinfold thickness (SISKF), body mass index (BMI) and body fat percentage (%BF), were determined. The differences between groups were performed accordingly to normal and non-normal distribution. Spearman’s correlations were performed to analyze the hypothetical correlation between KIDMED and BMI.

**Results:**

Players reached an average KIDMED score of 8.36 ± 1.92, showing that 68.2% (n = 90) reached high adherence to the MD, 31.1% (n = 41) had moderate adherence to the MD, and 0.78% (n = 1) had poor adherence to the MD. When the analysis was made for age group and BMI classification, no significant differences were observed in adherence to the MD. Considering the main characteristics of the MD, 50.8% consumed fruit (vs. 49,2%), 52.3% consumed vegetables (vs. 47,7%), and only 20% consumed oleaginous dried fruits (vs. 80%). Dairy consumption throughout the day was 49,2% (vs. no: 50,8%).

**Conclusion:**

Data from the present study showed that many soccer players adhered to the MD, and no differences were observed for age group or BMI classification.

## Introduction

Soccer is the most popular sport globally, played by more than 250 million players [1]. It is considered an intermittent team sport involving many low-intensity actions intercalated with frequent high-intensity activities, such as accelerations and decelerations, rapid changes in directions, jumping, and landing tasks [2, 3]. Also, physical contact between opponents to gain or keep possession of the ball is typical during a soccer match, constantly altering the energy requirements to perform and recover [4].

In the earliest stages of their development, children and adolescents may benefit from an increased energy intake to meet the growth, development and maturation requirements [5]. Beyond the genetic components, regular physical activity also contributes to the proper functioning and development of the body during childhood [6]. On the other hand, high intensity-exercise training together with a deficit in nutritional intake can lead to a delay in the onset of puberty, bone health problems, development of unbalanced eating behaviors, increased risk of injury, short stature or menstrual irregularities in the case of girls [5, 7–9].

The nutritional requirements of an athlete, such as energy intake, the optimal number of macronutrients, micronutrients and fluids, vary according to age, sex, performance level, type of sport and body mass [10, 11].

Considering the association between diet and exercise that impacts athlete’s performance and recovery, recommendations for optimal diet have emerged [1, 12, 13]. Notably, the Mediterranean Diet (MD) has been recognized as an optimal nutrition option with a wide range of benefits in health [14–16]. MD was described for the first time by Ancel Keys in the 60’s when describing the eating pattern of the countries bathed by the Mediterranean Sea and is characterized by a significant consumption of fresh vegetables and fruits and with minimum processed foods ingestion [17]. In 2010, United Nations Educational, Scientific and Cultural Organization (UNESCO) classified this diet regimen as an Intangible Cultural Heritage of Humanity [14]. In Portugal, a team from the Faculty of Nutrition and Food Sciences of the University of Porto created the Mediterranean Food Wheel Guide, aiming to emphasise the wheel’s food aspect and the lifestyle associated with the Mediterranean Food Pattern [18]. Interestingly, MD is likely to affect sports performance [19, 20] positively. A randomized-sequence crossover trial showed that 4-days of MD improved endurance exercise performance in 11 recreationally active women (n = 7) and men (n = 4) (body mass index, 24.6 ± 3.2 kg/m^2^; age 28 ± 3 years) [19].

Body composition is an attribute of great importance since it has a solid link to physical performance and injury risk [4, 21–25]. Hereupon, assessing body composition of athletes [26, 27] and developing programs to improve and/or maintain body composition [28] were a current practices in sports.

Some studies already show a relation between adherence to the MD, better physical fitness and body composition in children and adolescents [29, 30] and a better quality of life [31]. Along the same line, we can find some studies addressing the level of adherence to the MD in youth [32–34]; however, this information on youth athletes is very scarce. Therefore, our aim was to characterize the adherence to the MD and analyze its association with anthropometric parameters in young soccer players.

## Methods

### Participants

**Fig. 1:**
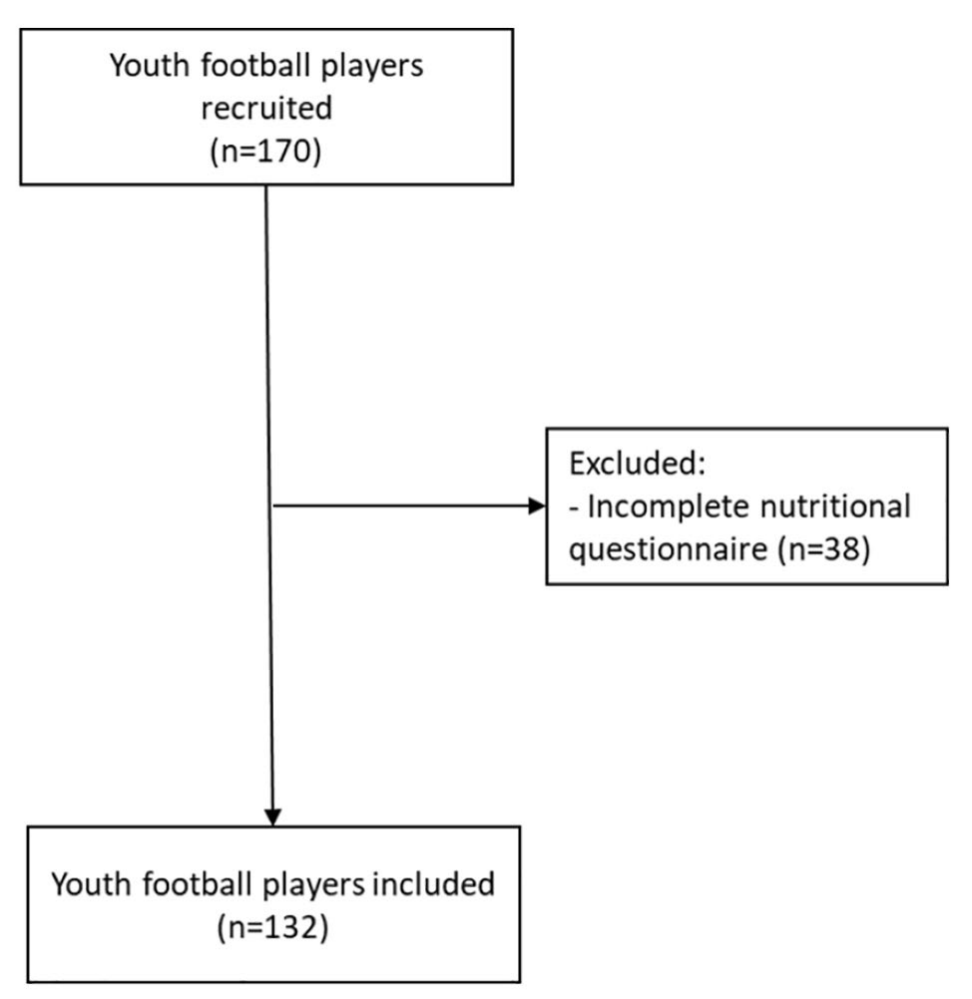
Flow diagram of the participants

In this cross-sectional study, 170 male soccer players from youngest categories (U9 to U15) were recruited, of which 132 completed the study [mean ± SD of the final sample; age: [12.0 ± 2.2 years], body weight: [48.35 ± 12.90 kg], height: [153.8 ± 14.90 cm], body fat percentage (%BF): [20.1 ± 5.2]]. Of all, 38 were excluded due to lack of response to the questionnaire (Fig. 1). The study was carried out during the competitive phase (April to May). This team was a convenience sampling for the ease access to nutritional and training/competition data by researchers.

All players and their legal guardians were informed about the research protocol, requisites, benefits, and risks and their written consent was obtained before the beginning of the study. Anonymity was preserved for all participants. The study was conducted according to the Declaration of Helsinki (revised version of 2013 at the 64th WMA General Assembly, Fortaleza, Brazil). The present study was approved by the Ethics Committee of the Instituto Universitário de Ciências da Saúde (14/CE-IUCS/2021).

The inclusion criteria were as follows: (i) soccer players U9 to U15 years; (ii) well-defined field position; (iii) legal guardians sign the informed consent; (iv) questionnaire correctly completed. In case the legal guardian did no sign informed consent or if the questionnaire has not been filled out completely, the player could not participate in the present study.

### Training sessions contextualization

The U9, U10, U12 and U13 categories undertook 3 training sessions per week with 90 min of duration per session, and a competitive match at the end of the week. Both U14 and U15 had 4 training sessions per week complemented with 2 gym training sessions and a competitive match at the end of the week (Table 1).

For all age groups, the training sessions were composed of integrated tactical, technical, and physical demands, with the incorporation of gym training from U15.

**Table 1:** Schematic of the typical training microcycle of athletes

	Monday	Tuesday	Wednesday	Thursday	Friday	Saturday	Sunday
U9	18:30–19:30 Pitch-based training	18:30–19:30 Pitch-based training		18:30–19:30 Pitch-based training		Match 11:00 (kick-off)	
U10	19:00–20:00 Pitch-based training	19:00–20:00 Pitch-based training	19:00–20:00 Pitch-based training			Match 09:00 (kick-off)	
U11	19:00–20:00 Pitch-based training	19:00–20:00 Pitch-based training	19:45–20:45 Pitch-based training			Match 09:00 (kick-off)	
U12	19:45–20:45 Pitch-based training	19:00–20:00 Pitch-based training		19:45–20:45 Pitch-based training		Match 13:00 (kick-off)	
U13	19:15–20:45 Pitch-based training		19:15–20:45 Pitch-based training	18:30–20:00 Pitch-based training		Match 15:00 (kick-off)	
U14		20:30–21:45 Pitch-based training	19:15–20:45 Pitch-based training	19:15–20:45 Pitch-based training	19:15–20:45 Pitch-based training		Match 11:00 (kick-off)
U15		19:30 − 20:25 Upper body gym session 20:30–21:45 Pitch-based training	18:15–19:10 Lower body gym session 19:15–20:45 Pitch-based training	19:15–20:45 Pitch-based training	19:15–20:45 Pitch-based training		Match 11:00 (kick-off)

### Evaluation of the Mediterranean diet quality index - KIDMED

To assess the level of adherence to the MD, the translated and validated Portuguese version of the KIDMED was applied over a week [35]. The questionnaires were given to the soccer players at the first training session of the week and filled out at home and then, delivered in the last training session of the week. This questionnaire is composed of 16 questions with YES or NO options. For each affirmative answer, question was scored with a + 1, except for questions 6, 12, 14 and 16 that were scored − 1 (Table 2). For negative answer, question was scored as -1. The total score of the KIDMED is the sum of all the items. The total score can vary between − 4 and 12 points, being attributed as “poor” adherence to the MD if ≤ 3 points, “moderate” to the MD 4 and 7 points and high when ≥ 8 points. All data collection was carried out by a registered dietitian.

**Table 2:** KIDMED Questionnaire to assess the adherence to Mediterranean diet quality index

	Question	Scoring
1	Drink natural fruit juice or eat a serving of fruit every day?	+ 1
2	Do you eat a second serving of fruit every day?	+ 1
3	Eat fresh or cooked vegetables once a day?	+ 1
4	Eat fresh or cooked vegetables more than once a day?	+ 1
5	Do you eat fish regularly, at least 2–3 times a week?	+ 1
6	Go once or more times a week to a fast-food restaurant (burger joint, pizzeria, etc.)?	-1
7	Eat legumes more than once a week (chickpeas, beans, lentils, peas, etc.)?	+ 1
8	Do you eat pasta, rice, bread, and potato almost every day (5 or more per week)?	+ 1
9	Have cereals or grains (bread, etc.) for breakfast?	+ 1
10	Do you eat nuts regularly, at least 2 or 3 times a week (walnuts, hazelnuts, almonds)?	+ 1
11	Use olive oil at home?	+ 1
12	Do you skip breakfast?	-1
13	Have a dairy product for breakfast (yogurt, milk, etc.)?	+ 1
14	Have commercially baked goods or pastries for breakfast?	-1
15	Consume two yoghurts and/or some cheese daily?	+ 1
16	Consume sweets and candy several times every day?	-1

### Anthropometric assessment

For anthropometric assessment, body mass, height and the measurement of 2 skinfolds (triceps and suprailiac, TSKF and SISKF, respectively) were measured by a certified International Society for the Advancement of Kinanthropometry (ISAK) level 1 expert, according to the standards accepted by the ISAK [36].

The body mass (kg) was measured on a digital scale (Smart Scale, Prozis, Braga, Portugal) to the nearest 0.01 kg. The height (cm) was evaluated using a portable stadiometer (SECA 213, Hamburg, Germany), with an approximation of 0.1 cm, with the participant’s head at the position of Frankfort’s horizontal plane. To assess the skinfolds, a calliper was used (Harpenden, British Indicators, Ltd., London, UK), with an approximation of 0.1 mm.

After the data was recorded, body mass index (BMI; kg/m^2^) was calculated by dividing body mass by height squared [37]. The percentage of body fat (%BF) was estimated by using the Lozano-Berges equation for two folds validated specifically for young football players [38] and, then calculated for age [39].

### Statistical analysis

The normality of the variables’ distribution was tested using the Shapiro-Wilk test. Mean ± standard deviation (SD) or median and interquartile range (IQR) were used to present descriptive statistics as data showed normal or non-normal distribution. The differences between groups were performed accordingly to normal and non-normal distribution. Spearman’s correlations were performed to analyze the hypothetic correlation between KIDMED and BMI and were classified as follows: negligible (0.0–0.1), weak (0.10–0.39), moderate (0.4–0.69), strong (0.70–0.89) and very strong (0.90–1.00) [40]. All analyses were conducted using the Statistical Package for Social Sciences software program (SPSS, version 27; IBM, Armonk, NY), and the alpha level was set a priori at 0.05.

## Results

The body mass was 48.81(19.7) kg, height was 153.8 ± 14.9 cm, BMI was 19.65(3.5), %BF was 20.1 ± 5.2, TSKF was 28.0 ± 11.1 mm, and the SISKF was 17.42 ± 19.8 mm. Differences were found in body mass, BMI, %BF, TSKF and SISKF between age groups, as depicted in Table 3. Generally, TSKF decreased in players U14 and U15 vs. players U13 and U12, while SISKF only decreased in U14 and U15 vs. U12.

**Table 3:** Anthropometric measurements of the total group and each group age

	Age years	Height cm		Body mass kg		BMI kg/m^2^	BF %	TSKF mm	SISKF mm
**Total** (N = 132)	11.98 ± 2.18	153.76 ± 14.86		48.80(19.70)		20.11(2.64)	20.12(5.24)	11.05(5.23)	17.42 ± 19.81
**U9** (N = 11)	7.82 ± 0.60 ^#$&¥¶º p<0.001^	129.64 ± 4.23 ^$ p<0.001^ ^& p<0.001^ ^¥ p<0.001^ ^¶ p<0.001^ ^º p<0.001^		29.10(5.50) ^$ p=0.003^ ^& p<0.001^ ^¥ p<0.001^ ^¶ p<0.001^ ^º p<0.001^		16.50(2.10) ^& p<0.001^ ^¥ p<0.001^ ^¶ p=0.004^ ^º p<0.001^	19.56 ± 3.86	10.46 ± 3.80	10.09 ± 5.49
**U10** (N = 15)	9.20 ± 0.41 ^#$&¥¶º p<0.001^	137.40 ± 6.54 ^& p<0.001^ ^¥ p<0.001^ ^¶ p<0.001^ ^º p<0.001^		35.20(4.4) ^& p=0.008^ ^¥ p<0.001^ ^¶ p<0.001^ ^º p<0.001^		18.40(3.0) ^º p=0.011^	21.20 ± 4.74	12.57 ± 5.15	10.08 ± 5.49
**U11** (N = 15)	10.40 ± 0.51 ^#$&¥¶º p<0.001^	143.73 ± 6.00 ^* p<0.001^ ^¥ p<0.001^ ^¶ p<0.001^ ^º p<0.001^		41.30(7.6) ^* p =0.003^ ^¥ p=0.28^ ^¶ p<0.001^ ^º p<0.001^		19.50(2.4)	22.82 ± 5.02 ^¶ p=0.023^ ^º p=0.016^	13.87 ± 5.17 ^¶ p=0.030^ ^º p=0.012^	13.23 ± 5.76
**U12** (N = 15)	11.33 ± 0.49 ^#$&¥¶º p<0.001^	149.20 ± 8.64 ^* p<0.001^ ^# p<0.001^ ^¶ p<0.001^ ^º p<0.001^		48.70(17.1) ^* p <0.001^ ^# p=0.008^ ^¶ p=0.014^ ^º p<0.001^		20.00(5.7) ^* p<0.001^	22.87 ± 8.25 ^¶ p=0.021^ ^º p=0.014^	13.03 ± 7.82	16.87 ± 12.61 ^¶ p=0.010^ ^º p=0.027^
**U13** (N = 22)	12.59 ± 0.50 ^#$&¥¶º p<0.001^	153.86 ± 6.36 ^* p<0.001^ ^# p<0.001^ ^$ p<0.001^ ^¶ p<0.001^ ^º p<0.001^		49.25(10.1) ^* p<0.001^ ^# p<0.001^ ^$ p=0.028^ ^º p<0.001^		20.25(3.4) ^* p<0.001^	22.28 ± 6.12 ^¶ p=0.022^ ^º p=0.014^	13.14 ± 6.06 ^¶ p=0.046^ ^º p=0.017^	13.39 ± 9.09
**U14** (N = 27)	13.48 ± 0.51 ^#$&¥¶º p<0.001^	164.15 ± 8.47 ^* p<0.001^ ^# p<0.001^ ^$ p<0.001^ ^& p<0.001^ ^¥ p<0.001^ ^º p<0.001^		53.0(10.6) ^* p<0.001^ ^# p<0.001^ ^$ p<0.001^ ^& p=0.014^ ^º p=0.025^		19.90(1.8) ^* p<0.001^	17.64 ± 2.44 ^$ p=0.023^ ^& p=0.21^ ^¥ p=0.22^	8.78 ± 2.82 ^$ p=0.030^ ^¥ p=0.046^	8.89 ± 4.08 ^& p=0.010^
**U15** (N = 27)	14.48 ± 0.58 ^#$&¥¶º p<0.001^	170.30 ± 7.17 ^* p<0.001^ ^# p<0.001^ ^$ p<0.001^ ^& p<0.001^ ^¥ p<0.001^ ^¶ p=0.41^		60.9(8.6) ^* p<0.001^ ^# p<0.001^ ^$ p<0.001^ ^& p<0.001^ ^¥ p<0.001^ ^¶ p=0.14^		21.0(1.8) ^* p<0.001^ ^# p<0.001^	17.46 ± 2.44 ^$ p=0.16^ ^& p=0.14^ ^¥ p=0.14^	8.35 ± 2.87 ^$ p=0.012^ ^¥ p=0.017^	9.57 ± 4.25 ^& p=0.027^

Data are expressed as mean ± SD or median (IQR). BMI: Body Mass Index; %BF: Percentage Body Fat; TSKF: tricipital skinfold; SISKF: suprailiac skinfold. *vs. U9; ^#^vs. U10; ^$^vs.U11; ^&^vs. U12; ^¥^vs.U13;^¶^vs.U14; ^º^vs.U15

**Fig. 2:**
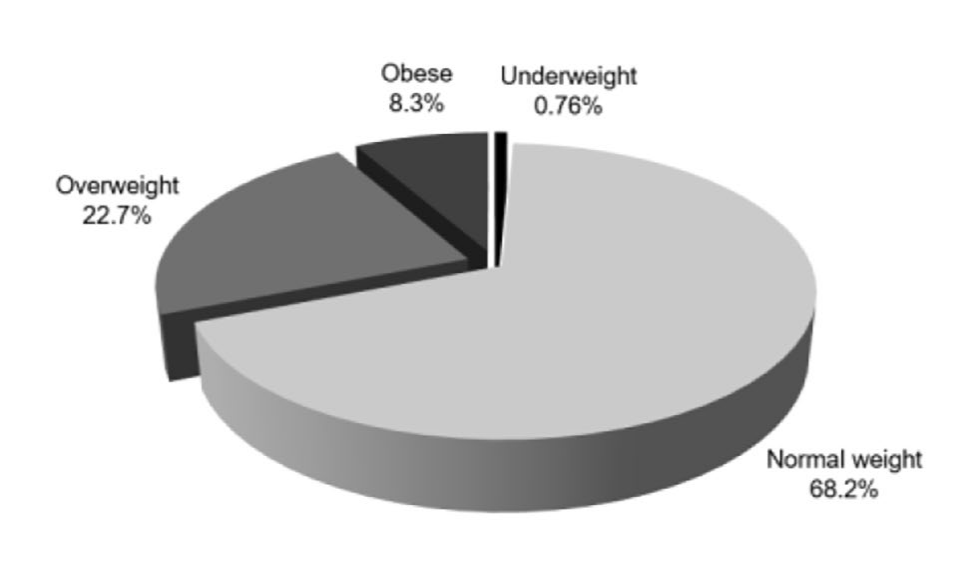
Absolute frequency of BMI classification

Based on BMI classification for each category [37], 68.2 (n = 90) of players were classified as average weight, 22.7% (n = 30) as overweight, 8.3% (n = 11) as obese and 0.76% (n = 1) as underweight (Fig. 2).

**Fig. 3:**
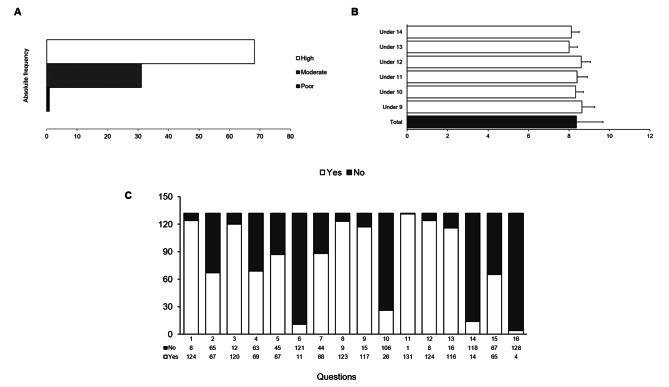
Level of adherence to the MD for the total sample (**A**), for each age group (**B**) and absolute frequency for each question (**C**) Data are expressed as mean ± SD.

The relative and absolute frequencies were obtained for the adherence index to the MD (Fig. 3). Players reached an average KIDMED score of 8.36 ± 1.92, showing that 68.2% (n = 90) reached high adherence to the MD, 31.1% (n = 41) had moderate adherence to the MD, and 0.78% (n = 1) have poor adherence to the MD (Fig. 3A). When the analysis was made for the age group, no significant differences were observed for the adherence to the MD (Fig. 3B).

**Fig. 4:**
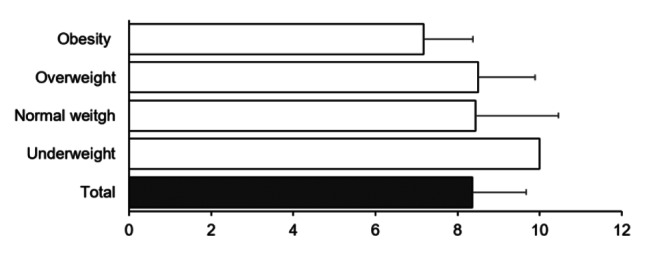
Adherence to the MD for BMI classification

The adherence to the MD between BMI classification groups was also analyzed, and no differences were observed (Fig. 4).

The score obtained from the KIDMED questionnaire was inversely correlated with BMI (r=-0.20; p = 0.19), namely for question 1 “Drink natural fruit juice or eat a serving of fruit every day?” (r = 0.22; p = 0.01), question 2 “Do you eat a second serving of fruit every day?” (r = 0.19; p = 0.03) and question 7 “Do you like and eat legumes (e.g., beans, peas, chickpeas, broad beans, lentils) more than once a week?” (r = 0.26; p > 0.01). Moreover, age inversely correlated with %BF (r=-0.24; p = 0.06) and TSKF (r=-0.24; p > 0.01).

## Discussion

Our study aimed to characterize adherence to the MD and analyze its association with anthropometric parameters in young soccer players. Data from the present study showed that a higher percentage of soccer players adhered to the MD, and no differences were observed for age group or BMI classification.

The physical and physiological requirements during soccer training and the match [1] diet is a very important factor influencing sports performance and recovery [10]. Several associations and groups discussed the role of diet and how athletic performance and recovery from training and match could be enhanced after optimal nutrition [1, 13, 41]. MD has been included in optimal nutrition for their benefits in health [42] and also represents an affordable sustainability model [43]. The utilization of the MD holds promise as a viable means for imparting knowledge about wholesome dietary practices and upholding dietary excellence among physically active adolescents who consistently partake in vigorous physical activities [44]. This diet regimen is characterized by high and varied consumption of fruits and vegetables, whole grains, extra virgin olive oil, nuts and seeds as the source of fat; moderate consumption of fish and dairy products and low consumption of red and processed meats [16].

Interestingly, MDs likely have positive effects on sports performance [19]. A randomized-sequence crossover trial showed that 4-days of MD improved endurance exercise performance in 11 recreationally active women (n = 7) and men (n = 4) (body mass index, 24.6 ± 3.2 kg/m^2^; age 28 ± 3 years) [19]. Hereupon, the level of adherence to the MD is of utmost relevance especially in the youngest ages, but it has been little explored. From the present study, a total of 132 soccer players were involved, and 31.1% achieved a moderate MD adherence index, and 68.2% reached a high rate of adherence to the MD, which is in accordance with findings from [45], also conducted in soccer players. In this line, studies [46, 47] carried out in Portugal showed that most children and adolescents from Lisbon and Viseu (n = 68; aged 6 to 16 years) reported moderate-to-high adherence to the MD. Children and adolescents (n = 276, aged 11 to 16 years) from the region of Algarve [47] reached higher adherence to the MD. In a reduced sample of soccer players (n = 33, aged 10 to 12 years) from North Portugal, 85% reported high adherence to the MD [34]. Altogether, these studies conducted in Portugal suggest that children and youth adhere at a higher level to the MD, contesting other studies [33, 48] shown in other countries that report low adherence to the MD.

Regarding anthropometric measurements, data obtained in the present study are similar to those obtained in other studies [34, 35]. Concerning the %BF, values of the present study were 20.12 ± 5.2 against 17.61 ± 6.3 from Spain and 8.26 ± 3.6 from Brazil, which is expected as each country has different levels of athletes. For instance, other studies [49] used a semiprofessional or professional male soccer player, unlike us. Moreover, using different equations for %BF prediction contributes to bias [50].

The sample of the present study aged between 7 and 15 years old and, thus, we tested the level of adherence to the MD for age group (U9, U10, U11, U12, U13, U14, U15) and each BMI category (underweight, average weight, overweight and obesity). No differences were observed for these two factors, which may be explained by the Mediterranean dietary pattern in Portugal [51] and the location where the study was carried out a smaller hinterlander. The city - Paços de Ferreira - is far away from big cities, as observed by question #6, in which 92% (n = 121) of the participants didn’t go once or more times a week to a fast-food restaurant, maintaining a more traditional food pattern.

Analyzing the questions #2: “Do you eat a second piece of fruit every day?” and #5: “Do you eat fresh vegetables (e.g., salad) or cooked vegetables (e.g., vegetable soup) more than once a day?”, almost half of the athletes (49.2% and 47.7%, respectively) reported a negative response. According to the Mediterranean Food Wheel Guide [52], 3 to 5 portions of fruits and vegetables groups are recommended [52] for their richness in vitamins, minerals and fiber. Moreover, fruits and vegetables have a high nutritional density and low energy density, which impact anthropometric measurements, and so an excellent nutritional option [53]. In this line, we observed an inverse association between BMI and question #2. Moreover, 31% of the participants were classified as overweight and obese, which may be explained in part by the low adherence to the second serving of fruit per day. After this diagnosis, information counseling about specific recommendations on this type of food and/or strategies to increase their ingestion should be provided to these young individuals.

Another question in which the answers do not meet the recommendations is #10, “Do you eat nuts (e.g., walnuts. almonds. hazelnuts) regularly (at least 2 to 3 times a week)?” which received a negative response from most participants (80.3%, n = 1065). The importance of nuts is related to their benefits of cardiovascular health due to their richness in both monounsaturated and polyunsaturated fats, vitamins, minerals, fibre and their antioxidant characteristics [54]. Although the consumption of nuts was low, the consumption of olive oil (question #11; 99%, n = 131). Olive oil is the traditional “figure” of the MD, representing the primary source of fat,. Their positive effects have been demonstrated in some pathologies, such as cardiovascular diseases/coronary heart disease, metabolic syndrome, diabetes mellitus type 2, breast cancer [55–58]as well as in weight control [59].

To question #15: “Consume two yoghurts and/or some cheese daily?”, approximately 51% (n = 67) of the participants reported not consuming these amounts of dairy products. The daily recommendations of the dose of dairy products (40 g of cheese or 1.5 yoghurt) [18] are not achieved by half of our sample. Dairy product consumption during childhood and adolescence lead to a higher peak bone mass [60]. Data from a systematic review revealed that 16 months of dairy products in various quantity increase 8% of bone mineral density during childhood and adolescence [61], contributing to the greater amount of bone mass at the end of skeleton maturation process and likely preventing osteoporotic fractures at a later stage of the life cycle [60]. Moreover, a higher gain in lean mass has also been related to the dairy products ingested [62], improving anthropometric-related parameters.

### Limitations

The present study comprises some limitations: (i) the questionnaire was self-applied (how its administration is recommended), which may lead to some errors due to memory bias; (ii) we did not use a 24-hour questionnaire to analyze the actual food pattern. In that sense, it is not possible to compare the answers to KIDMED and the actual food consumption; (iii) lack of data regarding the maturation level of the participants, and (iv) the information obtained by KIDMED is validated to assess diet quality, and it does not inform about the amount of food ingested. As a typical limitation of this kind of questionnaire, it would be interesting to enrich this kind of data with other quality and quantity food records combined with the KIDMED.

## Conclusions

Data from the present study demonstrates that most children and youth soccer players have a high adherence to the MD, suggesting a high-quality dietary pattern. In addition, a sample composed of young soccer players shows that it is necessary to prioritize food education for a more excellent perception of the short and long-term benefits of a Mediterranean Diet.

The significance of the Mediterranean diet (MD) pattern in attaining a nutritionally rich diet that caters to the growth, well-being, and athletic performance of adolescent athletes is paramount. Furthermore, educational interventions should endeavor to furnish athletes and coaches with knowledge about healthful approaches for attaining an optimal body weight specific to their sport and the detrimental consequences associated with adopting unhealthy and extreme dietary tactics.

For future studies, it would be interesting to complement the level of adherence to the MD with quantitative data (i.e., amounts of food ingested) and to evaluate longitudinally the level of adherence to the MD and the anthropometric parameters at the youngest ages. An evaluation of physical performance should also be considered in future studies to understand whether this diet has an advantage in sports performance.

## Data Availability

All data generated or analyzed during this study are included in this published article and their supplementary information files.
